# Magnitude, pattern and management outcome of intestinal obstruction among non-traumatic acute abdomen surgical admissions in Arba Minch General Hospital, Southern Ethiopia

**DOI:** 10.1186/s12893-021-01294-0

**Published:** 2021-06-15

**Authors:** Mulatie Atalay, Abinet Gebremickael, Solomon Demissie, Yonas Derso

**Affiliations:** 1 Department of Anatomy, Arba Minch University, Arba Minch, Ethiopia; 2 Departments of Physiology, Arba Minch University, Arba Minch, Ethiopia

**Keywords:** Magnitude, Obstruction, Management, Outcome, Arba Minch, Ethiopia

## Abstract

**Background:**

Intestinal obstruction is defined as a blockage or partial blockage of the passage of the intestinal contents. It is a potentially risky surgical emergency associated with high morbidity and mortality. Its pattern differs from country to country and even from place to place within a country. Therefore, this study aimed to find out the magnitude, pattern and management outcome of intestinal obstruction in Arba Minch General Hospital.

**Methods:**

A retrospective cross-sectional study was conducted in Arba Minch General hospital from January 09, 2015, to November 09, 2018. The data collection period was from December 15, 2018, to February 09, 2019. A simple random technique was applied to select 801 study participants. Then, the required data entered into Epi Info version 7.2.1.0 and exported to the statistical package for the social sciences software package version 20 for analysis. The binary logistic regression analysis has been done to determine crude statistical associations between independent variables and dependent variables. Linearity, Multivariate normality and multicollinearity were checked between independent and dependent variables by using scatter plot and Q–Q plot respectively. Variables with a p-value of less than 0.25 in the binary logistic regression analysis were entered into multivariable logistic regression. Statistical significance factors were identified based on a p-value of < 0.05 and with a 95% confidence interval.

**Result:**

This study revealed that the overall magnitude of intestinal obstruction was 40.60% with 95% CI (34.95–45.95). The magnitude of unfavorable management outcomes and deaths during the study period were 22.3% with 95% CI (18.00–27.00) and 7.1% with 95% CI (4.00–10.00) respectively. Persistent tachycardia 10.3 (3.28–32.42), Dehydration 13.7 (3.34–56.56), elevated serum creatinine 10.2 (1.89–54.94), gangrenous small bowel volvulus 2.7 (1.27–5.84), ischemic bowel 3.4 (1.17–9.81) and perforated bowl 7.68 (2.96–19.93) were significantly associated with the management outcome of intestinal obstruction.

**Conclusion and recommendation:**

Intestinal obstruction was the most common among all acute abdomen cases and its management outcome highly associated with dehydration. Adequate early preoperative resuscitation and proper post-operative care with appropriate surgical techniques and wound care with sterile techniques would help to reduce further mortality. This could be achieved by increasing public awareness of health-seeking behavior. Moreover, health facilities capable of handling patients with small bowel obstruction should be available within the reach of the community.

## Background

Intestinal obstruction (IO) is defined as a blockage or partial blockage of the passage of the intestinal contents. It is a potentially risky surgical emergency associated with high morbidity and mortality [1]. It is a frequently encountered surgical emergency that requires prompt diagnosis as well as immediate, rational and effective therapy [2]. It constitutes a major cause of death and financial expense in hospitals around the world and a major cause of admissions to emergency surgical units [3–5].

In a global based report of the world health organization, about 3.2 million cases of bowel obstruction occurred in 2015 which resulted in 264,000 deaths [6]. Both sexes are equally affected and the condition can occur at any age [7]. In most of the countries of Africa, it accounts for a significant proportion of morbidity and mortality which varies from region to region. For example, in Southwestern Nigeria, obstructed hernia [8], in Kenya, sigmoid volvulus [9], in Benin mechanical bowel obstruction [10] and strangulated hernia (particularly inguinal hernia) remains the most common cause of intestinal obstruction in tropical African populations [11].

Studies conducted in Ethiopia showed that the death rate after the management of intestinal obstruction cases were 13.6%, 9.2%, and 2.5% in South, East and Central Ethiopia respectively [12–14]. In studies conducted in Debre Birhan [15], in Gondar [16] and Mekele [17] showed that the magnitude of intestinal obstruction was higher than other non-traumatic acute abdominal surgical cases.

Aetiologies of bowel obstruction include sigmoid volvulus, small bowel volvulus, adhesions, hernias, inflammatory bowel disease, appendicitis, tumors, diverticulitis, ischemic bowel, tuberculosis and intussusception [18]. The causes of obstruction are different depending on the site of obstruction and between different areas [19]. A study was done in Uganda [20], Nigeria [21] and Ethiopia [22] shows that hernia (40.2%), Adhesions (51.6%) and small bowel volvulus (48.6%) were the leading causes of intestinal obstruction respectively.

Analysis of cases based on the specific causes of the acute abdomen has great value for early diagnosis and prompt treatment in clinical practice [23]. Despite the high prevalence of intestinal obstruction, there is a paucity of data concerning the magnitude and management outcome in Ethiopia [13, 16, 22, 24]. This study fills a gap of information on the magnitude, pattern and management of intestinal obstructions. It serves as an essential input for policymakers, medical students, surgeons, physicians and other health professionals to properly address IO. Furthermore, this will be used as baseline data for other investigators who are going to work on related issues.

## Methods and materials

### Study design, setting and population

A hospital-based retrospective cross-sectional study was conducted in Arba Minch General Hospital from January 09, 2015, to November 09, 2018. All patients with the diagnosis of non-traumatic acute abdomen cases who were admitted to the surgical ward of Arba Minch General hospital were included and patients who went home before completion of treatment, with lost cards and cards with incomplete data were excluded.

### Sample size determination

The sample size was determined by the single population proportion formula. The assumptions considered to calculate the sample size were from the previous study in Ethiopia using a prevalence of 21.8% [13]. Considering the precision 3% and 95% confidence level. Using the above assumptions, we arrived at a sample size of 728 and by adding 10% for loss of data; the final sample size becomes 801.

### Sampling producer

A total of 5590 total surgical admissions were found during the study period. Among these, 1303 of them were admitted with conditions attributed to non-traumatic acute abdomen. During the procedure, the medical record numbers were sorted from smallest to largest code (1–1303). The required sample size was obtained by using a simple random sampling technique. So, 801 cards were selected randomly from 1303 cards. But, among the sample size, only 761 cards were found with complete data. Cards/Medical records with incomplete data were excluded from the study.

### Study variables

#### Dependent variables

Management outcome of IO (favorable or unfavorable).

#### Independent variables

Socio-demographic characteristics (age, sex and residence), patient history (history of the previous operation, rectal bleeding, constipation, obstipation, and duration of illness, comorbidity (hypertension, diabetes, cardiac disease, renal condition and chronic liver disease), Physical examination findings [Vital sign derangement (persistent tachycardia, fever, dehydration), Blood on digital rectal exam], intraoperative findings (gangrenous small bowel volvulus, gangrenous sigmoid volvulus, adhesion and bands, viable small bowel volvulus, viable sigmoid volvulus, intussusceptions, tumor, strangulated hernia, viable hernia), intraoperative procedure (resection and anastomosis, adhesion release, manual reduction, derotation and diversion/stoma).

### Data collection procedure

The data were collected by using a structured checklist. The checklist was adapted from previous studies to enable us to collect important information. A checklist was developed in the English language to collect important information such as age, sex, ethnicity, admission clinical diagnosis, intraoperative findings, intra-operative procedures, duration of the presentation, causes of IO, postoperative complications and management outcome. For data collection, two clinical nurses were recruited.

### Operational definitions

Acute abdomen: a patient’s case which is labeled in their discharge diagnosis as one of the patterns of acute abdomen. Intestinal obstruction: a patient with acute abdomen who has abdominal pain, abdominal distension, vomiting, not passing gas and feces (obstipation) completely, not passing feces (constipation), with imaging diagnosis having obstructed dilated bowel loops and labeled in their discharge diagnosis note as intestinal obstruction. Management outcome: the condition of the patient after treatment has been carried out, after conservative or operative procedure that means whether discharged alive or died in the hospital or end up with complications. Favorable management outcome: discharged live and with no complication after management. Unfavorable management outcome: defined as a patient with intestinal obstruction developing one or more postoperative complications (including wound infection, fascial dehiscence, anastomotic leakage, developed septic shock, pelvic collection and pneumonia) and/or death.

### Data management and statistical analysis

Data were cleaned, coded and entered into Epi-info version 7.2.1.0 and was exported to SPSS version 20 for analysis. Descriptive statistics were conducted and results have been presented using frequency tables, graphs and percentages. The binary logistic regression analysis has been done to determine crude statistical associations between independent variables and dependent variables. Linearity between independent and dependent variables was checked by scatter plot. Multivariate normality between variables was checked by a Q–Q plot. And, multicollinearity was checked. Variables with a p-value of less than 0.25 in the binary logistic regression analysis were considered as a candidate to be entered into multivariable logistic regression. Multivariable analyses have isolated independent predictors of IO management outcome. Statistical significance factors were identified based on a p-value of < 0.05 with a 95% confidence interval.

## Results

**Fig. 1:**
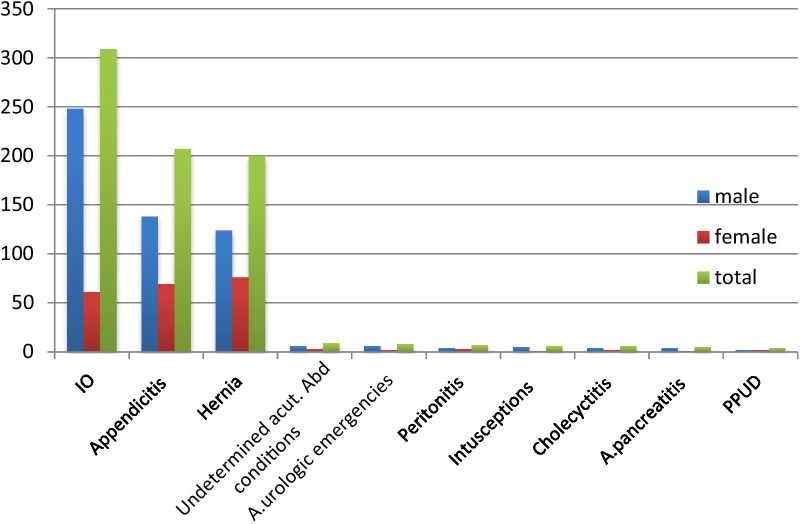
Bar graph of the pattern of non-traumatic acute abdomen in Arba Minch General hospital from January 09, 2015 to November 09, 2018

From the total study participants of 801, only 761 cards have complete data with a response rate of 95.12% where 507 (66.6%) cases were male, 478 (62.80%) were from a rural area (out of Arba Minch city) and 680 (85.42%) were less than 50 years old age. Among these 309 (40.60%) cases were found to have an intestinal obstruction which is the most common condition followed by Appendicitis 207 (27.20%), Hernia 200 (26.28%), ill determined acute abdomen conditions 9 (1.18%), urologic emergencies 8 (1.05%), peritonitis 7 (0.91%), intussusceptions, 6 (0.79%), cholecystitis 6 (0.78%), acute pancreatitis 5 (0.65%) and PPUD 4 (0.52%) respectively. Most of the cases from each were males (Fig. 1).

### Socio-demographic characteristics of intestinal obstruction cases

Of 309 cases with intestinal obstruction, 248 (80.25%) of them were males and 61 (19.74%) of them were females. The male:female ratio was 4:1. Among these, 243 (78.6%) of them were from a rural area (out of Arba Minch city), 64 (20.7%) of them were from urban (Arba Minch city) and 2 (0.6%) were with no information of residence. The age of patients ranged from 2 months to 100 years with a mean and standard deviation of (40.68 ± 17.88). Among these study participants, 195 (63.1%) were Protestants and 249 (80.9%) were Gamo in ethnicity (Table 1).

**Table 1:** Socio-demographic characteristics of patients with intestinal obstruction from January 09, 2015, to November 09, 2018, in Arba Minch General Hospital

Variables	Frequencies	Percent (%)
Sex		
Male	248	80.25
Female	61	19.74
Age (years)		
< 50	208	67.31
≥ 50	101	32.68
Residence		
Rural	243	78.64
Urban	64	20.71
Not known	2	0.64
Religion		
Protestant	195	63.10
Orthodox	108	34.95
Muslim	3	0.97
Others	3	0.97
Ethnicity		
Gamo	249	80.58
Konso	27	8.73
Amhara	23	7.44
Oromo	8	2.58
Others	2	0.67

### Magnitude and types of intestinal obstructions

The magnitude of intestinal obstruction was 40.45% with 95% CI (34.95–45.95) among non-traumatic acute abdominal cases. Of the total 309 intestinal obstructions, 302 (97.7%) of them were mechanical intestinal obstructions and the remaining 7 (2.3%) were a dynamic ileus/functional intestinal obstruction. According to the site of obstruction, 198 (64.1%) were small bowel obstructions, 108 (35.0%) were large bowel obstructions and the rest 3 (1.0%) of them were undetermined.

### Clinical presentation and diagnosis

The common clinical presentations were abdominal pain (95.8%), abdominal distension (88.0%), obstipation (78.3%), constipation (69.6%), vomiting (62.1%), rectal bleeding (2.6%) and history of abdominal surgery (7.1%). Dehydration, Persistent tachycardia, fever and shock were also seen in 5.8%, 10.7%, 12.6% and 2.6% of patients respectively. Hypertension, known cardiac disease, diabetic, CLD and CKD found in 4.9%, 1.3%, 1.9%, 1.3% and 1.3% of patients respectively. By laboratory diagnoses: hypokalaemia, leucocytosis, elevated serum urea, hyperkalaemia and elevated serum creatinine were found in 1.0%, 21.0%, 2.6%, 0. 3% and 3.9% of patients respectively.

### Duration of symptom, hospital stay and management approach

The time of arrival since the onset of disease was greater than 24 h for 253 (81.9%) of patients and less than or equal to 24 h for 56 (18.1%) of patients. The total hospital stays recorded was > 7 days for 170 (55.0%), < 7 days for 134 (43.41%) and equal to 7 days for 5 (1.6%) of patients. 63 (20.4%) of patients were managed only conservatively and 246 (79.6%) were managed surgically.

### Operative procedures, operative findings and complications

From the operative procedure undertaken resection and anastomosis 118 (38.2%) were the most common procedure done (Table 2).

**Table 2:** Procedures were done for intestinal obstruction patients in Arba Minch General hospital from January 09, 2015, to November 09, 2018

Procedures done	Frequency	Percent
Not operated	63	20.4
Operated		
Resection and anastomosis	118	38.18
Derotation	60	19.41
Adhesion release	29	9.38
Reduction	25	8.09
Diversion/stoma	15	4.85

Among the operative findings supposed to cause intestinal obstruction, small bowel volvulus was the most common followed by sigmoid volvulus, adhesion and bands, intussusceptions, appendicitis, hernia and tumor obstruction respectively (Table 3).

**Table 3:** Operative findings and their percentage among intestinal obstruction cases in Arba Minch General hospital from January 09, 2015, to November 09, 2018

Variable	Frequency	Percent (%)
Perforated bowel	54	17.47
Ischemic bowel	44	14.23
Gangrenous SV	34	11.00
Gangrenous SBV	103	33.33
Appendicitis	14	4.53
Intussusceptions	15	4.85
Strangulated hernia	10	3.23
Viable hernia	3	0.97
Viable SV	32	10.35
Viable SBV	37	11.97
Adhesion and bands	37	11.97
Tumor obstructions	11	3.55

**Fig. 2:**
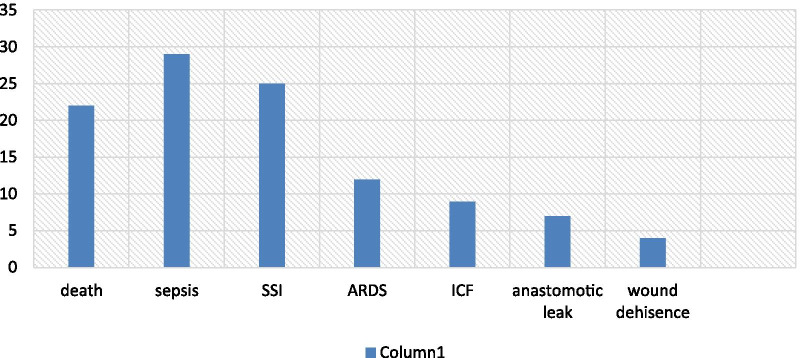
Bar graph of the pattern of complications after the management of intestinal obstruction from January 9, 2015, to November 9, 2018

Among operatively managed patients, 22.3% were developed complications and 7.1% of deaths occur throughout the study period. Sepsis and septic shock 29 (9.4%) were the most common complications. Surgical site infections 25 (8.1%), acute respiratory diseases 12 (3.9%), enter cutaneous fistula 9 (2.9%), anastomotic leakage 7 (2.3%) and wound dehiscence 4 (1.3%) were the remaining common complication types found respectively (Fig. 2).

### Association between factors and management outcome of intestinal obstruction

In the binary logistics regression dehydration, persistent tachycardia, elevated serum creatinine, gangrenous small bowel volvulus, ischemic bowl, shock, elevated serum urea and perforated bowl had an association with management outcome of intestinal obstruction (p-value < 0.25). However, only dehydration, persistent tachycardia, elevated serum creatinine, gangrenous small bowel volvulus, ischemic bowl and perforated bowl were significantly associated with the management outcome of intestinal obstruction in multivariable logistic regression (Table 4).

**Table 4:** Factors associated with management outcome of intestinal obstruction in Arba Minch General hospital from January 09, 2015, to November 09, 2018

Variables	Category	Frequency	Favorable management outcome	Unfavorable management outcome	COR 95% confidence interval	AOR 95% confidence interval
Persistent tachycardia	Yes	33	9 (27.27%)	24 (72.72%)	1	1
	No	276	231 (83.69%)	45 (16.30%)	13.68 (5.96–31.39)**	10.31 (3.28–32.42)**
Dehydration	Yes	18	5 (27.77%)	13 (72.22%)	1	1
	No	291	235 (80.75%)	56 (19.24%)	10.91 (3.73–31.86)**	13.73 (3.34- 56.56)**
Elevated serum creatinine	Yes	12	3 (25.00%)	9 (75.00%)	1	1
	No	297	237 (79.79%)	60 (20.2%)	11.85 (3.11–45.12)**	10.19 (1.89–54.94)*
Gangrenous SBV	Yes	103	60 (58.25%)	43 (41.74%)	1	1
	No	206	180 (86.53%)	26 (12.62%)	4.96 (2.81–8.75)**	2.72 (1.27–5.84)*
Ischemic bowel	Yes	44	15 (34.09%)	29 (65.90%)	1	1
	No	265	225 (84.90%)	40 (15.09%)	10.87 (5.36–22.08)**	3.39 (1.17–9.81)*
Perforated bowel	Yes	54	16 (29.62%)	38 (70.37%)	1	1
	No	255	224 (87.84%)	31 (12.15%)	17.16 (8.57–34.36)**	7.68 (2.96–19.93)**

*SBV* small bowl volvulus

**p < 0.001, *p < 0.05

## Discussion

The magnitude of intestinal obstruction in this study is 40.45% at 95%, CI (34.95–45.95) which is consistent with a result of a study at Gonder University Hospital (43.4%) [16]. But it is less than from the study conducted in Debre Birhan referral hospital (50.7% [15] and higher than from the studies of Nigeria (10%), Adama (21.8%) and Mekele (20.4%) respectively [13, 17, 25]. This discrepancy may be due to the difference in socio-cultural, economic and lifestyle patterns between countries or due to differences in statistical parameters including sample size.

The majority of intestinal obstruction cases were small bowel obstruction (64.1%) which is similar to the studies conducted in eastern and central Ethiopia [12, 13, 15, 22, 24]. Abdominal pain (95.8%), abdominal distension (88.0%) and obstipation (78.3%) are the most common clinical symptoms in our study. But this result is different from other studies in Ethiopia, where abdominal pain and abdominal distension covered 100% of cases in studies in eastern Ethiopia through [12, 13, 15, 22, 24]. The difference may be due to the small sample size.

The commonest causes of intestinal obstruction found from intraoperative findings were small bowel volvulus 45.30%, sigmoid volvulus 21.35%, adhesion and bands 12.0% and intussusceptions 4.9%. This result is similar to studies conducted in Gelemso and Chiro General Hospital [12, 24]. But, different from the study done in Adama Medical College Hospital and Debre Birhan Referral Hospital where intussusceptions (30.9%) and sigmoid volvulus were the leading findings respectively [13, 15]. These discrepancies may be due to the socio-demographic form of patient flow difference.

The magnitude of post-management complication in this study is 22.0% with 95% CI (18.0–27.0). This result is consistent with the studies conducted in Nigeria (20.77%), Kenya (23.6%) and Adama (24.6%) in Adama [19, 25, 26]. The magnitude of death over the study period is 7.11% with 95% CI (4.0–10.0). This result is almost similar to most of the findings of studies in other areas in Ethiopia [22, 24].

Sepsis and septic shock was the most common pattern of complication after management in the current study. This is different from the study in Gelemso General Hospital which found surgical site infection as the most common complication and Jogla General Hospital which found pneumonia as the most common complication type and septic shock next to it [12, 22]. This may be due to low care of the patient after management and longer hospital stay time.

In this study, patients without dehydration were 13.73 times more likely to have favorable management outcome, [AOR with 95% CI (13.73 (3.34–56.56))], than those with dehydration which is similar to the study conducted in Nigeria.

Persistent tachycardia is significantly associated with management outcome of intestinal obstruction, [AOR with 95% CI (10.31 (3.28–32.42))], in which patients without persistent tachycardia are 10.31 times more likely to have favorable management outcome than those with persistent tachycardia which is supported by a study conducted in Nigeria [27].

Elevated serum creatinine is significantly associated with management outcome of intestinal obstruction, [AOR with 95% CI (10.19 (1.89–54.94))], in which patients without elevated serum creatinine are 10.19 times more likely to have favorable management outcome when compared with those patients who have elevated serum creatinine. This may be due to the fact that in patients with renal function impairment creatinine levels may increase [28].

Perforated bowel, ischemic bowl and gangrenous small bowel volvulus are also significantly associated with management outcome of intestinal, [AOR with 95% CI (7.68 (2.96–19.93), 3.39 (1.17–9.81) and 2.72 (1.27–5.84))] respectively, in which patients without perforated bowl, ischemic bowl and gangrenous small bowel volvulus are 7.68, 3.39 and 2.72 times more likely to have favorable management outcome than their counterparts respectively. This result is similar to study findings conducted in East Ethiopia [22, 24].

## Limitations

Since this study was from secondary data, Limitations were incomplete documentation, missing charts and difficulty interpreting information found in patients' cards.

## Conclusions and recommendations

### Conclusions

In conclusion, intestinal obstruction was the most common pattern of non-traumatic acute abdomen conditions during the study period. Males were affected more than females. Intestinal obstruction was more common in rural residents. Small intestinal obstruction was more dominant than large bowel obstruction. Small bowel volvulus, sigmoid volvulus and adhesion and bands were the commonest patterns of intestinal obstruction respectively. Resection and anastomosis, derotation and adhesion removal were commonest procedures done respectively. Dehydration, persistent tachycardia, elevated serum creatinine, gangrenous SBV, ischemic bowl and perforated bowl were significantly associated with the management outcome of intestinal obstruction. Therefore, designing a strategy addressing these factors would be helpful to decrease the likelihood of unfavorable surgical management outcomes for the patients attending hospital with IO.

### Recommendation

Based on our findings we suggest that health professionals in the hospital should increase public awareness on IO by providing appropriate health information.

Physicians should diagnose and intervene on time before the intestine develops a complication.

Health professionals should give attention to appropriate surgical techniques and wound care with sterile techniques to decrease surgical site infection which is the most common complication.

Cardroom staff should improve record-keeping in the hospital because some medical records were incomplete.

Further research using a prospective study design is recommended as a way to overcome the limitations of secondary data in the current retrospective research that preclude generalization to the whole population.

## Data Availability

All relevant data are included in the article. The dataset of this study is available from the corresponding authors upon reasonable request.

## Abbreviations

ABO: Acute bowel obstruction
AOR: Adjusted odd ratio
ARDs: Acute respiratory diseases
CKD: Chronic kidney disease
CLD: Chronic liver disease
COR: Crude odd ratio
DALYs: Disability adjusted life years
ICF: Inter cutaneous fistula
GBDs: Global Burden of Diseases
HICs: High-income countries
IO: Intestinal obstruction
IV: Intra venous
LMICs: Low and middle-income countries
LBO: Large bowel obstruction
PID: Pelvic inflammatory disease
PPUD: Perforated pelvic ulcer disease
SBO: Small bowel obstruction
SBV: Small bowl volvulus
SSI: Surgical site infections
SV: Sigmoid volvulus
YLL: Years of life lost

## References

[1] Ullah S KMIo. A spectrum of causes, Department of Surgery, Postgraduate Medical Institute Lady Reading Hospital, Peshawar Pakistan. JPMI. 2008;8(1):210–3.

[2] Díte PLJ, Novotný I (2003). Intestinal obstruction and perforation– the role of the gastroenterologist. Dig Dis.

[3] Miller GBJ, Shrier I, Gordon PH (2000). Etiology of small bowel obstruction. Am J Surg.

[4] Miller GBJ, Shrier I, Gordon PH (2000). Natural history of patients with adhesive small bowel obstruction. Br J Surg.

[5] Mucha P (1987). Small intestinal obstruction. Surg Clin N Am.

[6] WHO (2015). GBD 2015 Disease and injury incidence and prevalence. Lancet.

[7] Ferri FF. Ferri’s clinical advisor books in 1. Elsevier health sciences. 2015;5:1093.

[8] Shittu OB, Gana J, Alawale EO, Ogundiran TO (2001). The pattern of mechanical intestinal obstruction in Ibadan. Afr J Med Sci.

[9] Muyembe VM, Suleman N (2000). Intestinal obstruction at a provincial hospital in Kenya. East Afr Med J.

[10] Chiedozi LC, Aboh I, Piserchia NE (1980). Mechanical bowel obstruction. Am J Surg.

[11] Adesunkanmi ARKAE (2011). Changing pattern of acute intestinal obstruction in a tropical African population. East Afr Med J.

[12] Gudina E, Abdifatah DS. Intestinal obstruction surgical management outcome and associated factors in Gelemso General Hospital, Oromia Regional State, Eastern Ethiopia. Haramaya University. 2016.

[13] Soressa U, Mamo A, Hiko D (2016). Prevalence, causes and management outcome of intestinal obstruction in Adama Hospital, Ethiopia. BMC Surg.

[14] Demissie M (2001). Small intestinal volvulus in southern Ethiopia. East Afr Med J.

[15] Yohannes M, Fanta M, Molla T (2017). The proportion of intestinal obstruction and associated factors among patients with non traumatic acute abdomen admitted to surgical ward in Debre Birhan Referral Hospital, North East Ethiopia. Am J Biomed Life Sci.

[16] Tsegaye S, Osman M, Bekele A (2007). Surgically treated acute abdomen at Gondar University Hospital, Ethiopia. East Cent Afr J Surg.

[17] Berhane Y, Girmay K, Gebresilassie A (2016). Out come of emergency surgical operations performed for nontraumatic acute abdomen among adults in Mekelle Hospital, Tigray, Ethiopia. Eur J Pharm Med Res.

[18] Gore RS, Silvers RI, Thakrar KH, Wenzke DR, Mehta UK, Newmark GM, Berlin JW (2015). Bowl obstruction. Radiol Clin N Am.

[19] Soressa U, Mamo A, Hiko D, Fentahun N (2016). Prevalence, causes and management outcome of intestinal obstruction in Adama Hospital, Ethiopia. BMC Surg.

[20] Okeny PK, Hwang TG, Ogwang DM. Acute bowel obstruction in a rural hospital in Northern in Northern Uganda. East Cent Afr J Surg. 2011;16(1).

[21] Ojo EO, Ihezue CH, Sule AZ (2015). Aetiology, clinical pattern and outcome of adult intestinal obstruction in Jos, North Central Nigeria. Afr J Med Med Sci.

[22] Ali Ge G, Sertse E. Intestinal obstruction surgical management outcome and associated factors in Jogla General Hospital, Harari Regional State, Eastern Ethiopia. Haramaya University. 2018.

[23] Asefa Z (2000). Pattern of acute abdomen in Yirgalem Hospital, southern Ethiopia. Ethiop Med J.

[24] Said S, Mahamud M. Intestinal obstruction surgical management outcome and associated factors in Chiro Zonal Hospital, Oromia Regional State, Eastern Ethiopia. Haramaya University. 2016.

[25] Bankole AO, Osinowo AO, Adesanya AA (2017). Predictive factors of management outcome in adult patients with mechanical intestinal obstruction. Niger Postgrad Med J.

[26] Gachini JMPTKR. Pattern, management and outcome of intestinal obstruction at the Moi Teaching and Referral Hospital. Moi University. 2015.

[27] Bankole AO, Osinowo AO, Adesanya AA. Predictive factors of management outcome in adult patients with mechanical intestinal obstruction. NPMJ. 2018:196. 10.4103/npmj.npmj_143_17 29355160

[28] Nankivell BJ. Creatinine clearance and the assessment of renal function: Australian Prescriber; 2001.

